# CNS minimal disease therapy in childhood leukaemia: the place for irradiation.

**DOI:** 10.1038/bjc.1990.218

**Published:** 1990-07

**Authors:** O. B. Eden

**Affiliations:** Royal Hospital for Sick Children, Edinburgh, UK.


Br. J. Cancer (1990), 62, 6–7 © Macmillan Press Ltd., 1990

GUEST EDITORIAL

### CNS minimal disease therapy in childhood leukaemia: the place for irradiation

O.B. Eden

*Royal Hospital for Sick Children, Sciennes Road, Edinburgh EH9 1LF, UK.*

In the 1960s, when the initial trials of combination chemotherapy began to prolong remission duration in childhood acute lymphoblastic leukaemia, an increasing incidence of central nervous system relapse was noted with rates between 50 and 70% (Hardisty & Norman, 1967; Evans *et al.*, 1970; Hustu *et al.*, 1973). Overt CNS leukaemia appeared difficult to control, but its major significance was that it was almost invariably followed by bone marrow relapse, whilst deaths from neurological dysfunction were quite rare, even in the presence of multiple CNS recurrences (Ortega *et al.*, 1987). Symptom-free remission post overt CNS relapse was short and, in the MRC trials between 1977 and 1987, only 23 of 116 children survived without subsequent event. The length of first remission and whether relapse occurred on or off therapy significantly determined the chance of survival. There were serious long-term sequelae with 50% of survivors severely disabled neurologically, educationally or on both counts. A more favourable salvage rate of over 50% has been reported by the Paediatric Oncology Group but it is in a series with a shorter follow-up time (Land *et al.*, 1985). The full impact of disability caused by therapy requires long-term follow-up.

Prevention of CNS relapse has clearly been deemed a better option than salvage post relapse. Although for some time controversial it is now accepted that CNS involvement results from leukaemic cells lying in the walls of superficial arachnoid veins which then migrate into the surrounding adventitia. As cell numbers increase the arachnoid trabeculae are destroyed with subsequent penetration of cerebrospinal fluid channels over the surface of the brain. Further extension can lead to direct infiltration of neural tissue. Rarely, isolated nodular infiltrates can occur. The leukaemic cells lying adjacent to the arachnoid veins are not reached by adequate concentrations of standard dose leukaemic therapy, that is, they lie in a so-called pharmacological sanctuary. Pinkel and his group at St Jude's Childrens Research Hospital postulated and then demonstrated that presymptomatic CNS irradiation could dramatically reduce overt CNS relapse to less than 10% (Aur *et al.*, 1971; Hustu *et al.*, 1973). Whether such treatment prevents subsequent haematological relapse is more controversial. The Cancer and Leukaemia Group B in a randomised study showed a median remission duration of 26 months for those receiving intrathecal methotrexate compared with only 15 months for the group which did not (Holland & Glidwello, 1972). In the St Jude's series of studies, there was a 2–3.5-fold increased risk of haematological relapse for those children who experienced an isolated CNS relapse. In the Total VI Study, 58% of patients who had received presymptomatic craniospinal irradiation remained in remission 8–11 years post-diagnosis, compared with only 35% of those given CNS treatment at the time of overt relapse (George *et al.*, 1985). In contrast, the CCGF101 study contained one arm in which patients were treated only with intrathecal methotrexate. This group had an increased CNS relapse rate, but neither marrow relapse nor overall survival rates were significantly affected after medium-term follow-up (Ortega *et al.*, 1987). However, these patients were recalled for further therapy when the increased CNS relapses became obvious. With increasingly intensive induction and consolidation therapy longer term follow-up (beyond 10 years) is necessary in order to assess the full impact of any overt CNS disease upon total survival. For some patients it would appear that occult CNS leukaemia is the only site of residual disease which, if not eradicated, will cause problems by recurring and eventually reseeding the marrow. For others it is a marker of more widespread disease already present. In those, central nervous system control with irradiation alone would merely lead to the initial relapse occurring in a non-CNS site.

Initially the use of craniospinal irradiation, although effective, was associated with excessive and unacceptable marrow ablation from the spinal component and the 'gold standard' therapy pioneered by the St Jude group was 24 Gy in 14–15 fractions over 17–18 days with intrathecal methotrexate, initially in a dose of 12 mg $m^{-2} \times 5$ given every 3–4 days. Radiation doses were scaled down for young children (20 Gy for 1-year-olds and 15 Gy for those $<1$ year). Higher fraction dosage, less fractions per week and longer duration of total therapy have not been shown to be advantageous. Modifications which do appear to have been worthwhile are the adjustment of methotrexate dosage by age which appears to correlate better with CSF volume than surface area and toxicity has been reduced by using weekly rather than twice weekly lumbar punctures without apparent loss of efficacy. The Children's Cancer Study Group (CCSG) have demonstrated that for those with an initial peripheral white count $<50 \times 10^9 l^{-1}$, 18 Gy is as effective as 24 Gy (Nesbit *et al.*, 1981). For those with higher initial white cell counts there was a trend for better CNS control with 24 Gy (of borderline statistical significance).

It is the toxicity of this therapy which has caused particular anxiety and led to approaches which avoid combined modality therapy, particularly CNS irradiation, whenever possible. Acute neurotoxicity during cranial irradiation is rare apart from somnolence syndrome (symptoms maybe lessened by concomitant steroids), but immediate post-radiotherapy severe immunosuppression, partial or complete growth hormone deficiency, premature onset of puberty and most significantly intellectual impairment are worrying sequelae from CNS therapy. Intellectual impairment is worst in children under 3 years at diagnosis and although they may function intellectually within the normal range they perform less well than siblings or peer group controls. Short-term memory appears to be specifically affected and this can be a major handicap in the achievement of basic numeracy and literacy (Eiser & Lansdown, 1977; Janoun, 1983). To date the reduction from 24 to 18 Gy does not appear to decrease this risk. In the long term there may be a correlation between neuropsychological changes and abnormalities reported on CT and MRI scanning (Brouwers *et al.*, 1985). Although periventricular hypodensities may disappear with time it may be years before calcification develops, and therefore very long-term assessment is required before reliable conclusions can be drawn on toxicity.

The search is thus on for a group of children in whom cranial irradiation can be avoided without jeopardy to their

Received 17 January 1990.

survival and conversely identification of those where there is no alternative. Green *et al.* (1980) retrospectively reviewed a number of studies comparing those given standard therapy with radiation and intrathecal methotrexate and those who either received a prolonged course of triple intrathecal chemotherapy only and those receiving intermediate dose systemic methotrexate plus intrathecal methotrexate. The lowest CNS relapse rates (reaching significance) were recorded for those given irradiation in both standard ($P<0.05$) and high risk categories ($P<0.001$), but the lowest haematological relapse rate for standard risk patients was achieved with systemic intermediate dose methotrexate. In the high risk patients the best relapse-free and total survival was obtained with irradiation. Where there was a high CNS relapse rate total survival was poorest, but where it was lowest it did not necessarily predict for overall good survival. The most important point appeared to be to achieve the best systemic leukaemia cell kill. This was a review and not a randomised concurrent trial and the systemic methotrexate dosages were low by current standards. High dose systemic therapy in dosages of $6-8\,g\,m^{-2}$ give cytocidal concentrations ($10^{-5}$ m) for 24–36 hours (Bleyer, 1989). Appropriately phased folinic acid rescues normal cells outside the CNS and possibly blasts there, but not those within the CNS provided the rescue is given when the CSF methotrexate concentration falls below the concentration cytotoxic to the leukaemic cells. Such therapy may not require the addition of intrathecal therapy. In a series of escalating dose studies the NCI and CCSG (Poplack *et al.*, 1984) have showed that for intermediate and poor risk patients such methotrexate therapy is as effective as 24 Gy cranial radiotherapy with standard intrathecal methotrexate. They have also investigated the relative toxicities of the different forms of CNS treatment. Those irradiated showed a steady decline in verbal IQ and in full scale IQ whilst those receiving methotrexate demonstrated a slight increase in both parameters with a marginal fall in results of arithmetic tests. Patients who had been irradiated showed a decline in reading, spelling and arithmetic tests (Brouwers, 1987). There must be some reservations about these observations. Follow-up is relatively short at 4–5 years and repeat testing can give spurious results. Most previous studies have suggested that post-irradiation verbal IQ is relatively spared and that the normal deterioration is in non-verbal performance.

Some questions remain and we cannot be absolutely confident that there will be no long-term sequelae from these high doses of systemic methotrexate, but the NCI/CCSG's studies included patients receiving dosages as high as $33\,g\,m^{-2}$. Repeatedly studies have demonstrated that the more modalities used for CNS minimal disease treatment the higher the risk of toxicity and that systemic methotrexate alone appears to be the least toxic, but still effective of the modalities. Intrathecal therapy with methotrexate alone may be marginally more toxic, but certainly less effective. There are strong suggestions from the POG group that triple intrathecal therapy with methotrexate, cytosine and hydrocortisone may be effective enough at preventing CNS relapse in at least low-risk patients. However, we have not good long-term evidence with regard to toxicity. The worst risk is undoubtedly the combination of radiotherapy, intrathecal methotrexate and systemic methotrexate, especially when given in that sequence. To date no group of children has been demonstrated to have no risk of overt CNS relapse, but there may be some with very low risk. In the Medical Research Council UKALL VIII Study where the majority of patients received 18 Gy plus six intrathecal methotrexate injections, but only those with initial white counts $>100 \times 10^9\,l^{-1}$ received 24 Gy, the isolated CNS relapse rate was 1.4% for girls aged 3–6 years with a white count $<10$ for all other patients with a white count up to $50 \times 10^9\,l^{-1}$ it was approximately 7% and above that white count 11–12% with a slight sex difference (Eden *et al.*, 1989).

In the future, we may be able to define those girls who truly do not need any CNS treatment, meanwhile it would seem reasonable to try to avoid irradiation in those with standard risk (white count up to $50 \times 10^9\,l^{-1}$) but use systemic methotrexate which may also afford some initial benefit in terms of overall disease-free survival. For the high risk patients matters are less clear, but a randomisation between irradiation and systemic methotrexate would appear justified. These form the basis of the next Medical Research Council ALL Trial with no change otherwise in the well established systemic treatment. The emphasis throughout must be upon total body leukaemia cell kill without overdue preoccupation with specific focal sites. High-dose systemic methotrexate in dosages between 6 and $10\,g\,m^{-2}$ seem to offer the chance of this without evidence to date of excessive toxicity. It is essential that such modern studies must incorporate assessment of toxicity.

### References

AUR, R.J.A., SIMONE, J.V., HUSTU, H.O. *et al.* (1971). CNS therapy and combination chemotherapy of childhood lymphocytic leukaemia. *Blood*, **37**, 272.

BLEYER, W.A. (1989). Proceedings of workshop on the role of clinical pharmacology in paediatric oncology. SIOP, Prague.

BROUWERS, P., RICCARDI, R., FEDIO, B. *et al.* (1985). Long term neuropsychologic sequelae of childhood leukaemia: correlations with CT scan abnormalities. *J. Paediatr.*, **106**, 723.

BROUWERS, P., MOSS, H., REAMAN, G. *et al.* (1987). Central nervous system preventive therapy with systemic high dose methotrexate versus cranial radiation and intrathecal methotrexate: Longitudinal comparison of effects of treatment on intellectual function of children with acute lymphoblastic leukaemia. *Proc. Am. Soc. Clin. Oncol.*, **6**, 158.

CHESSELLS, J.M., COX, T., CAVANAGH, N. *et al.* (1987). Methotrexate, cranial irradiation and neurotoxicity in childhood acute lymphoblastic leukaemia. *Proc. Int. Soc. Pediatr. Oncol.*, **16**, 102.

EDEN, O.B., LILLEYMAN, J.S., RICHARDS, S. *et al.* (1989). Results of Medical Research Council Childhood Leukaemia Trial UKALL VIII (in preparation).

EISER, C. & LANSDOWN, R. (1977). Retrospective study of intellectual development in children treated for acute lymphoblastic leukaemia. *Arch. Dis. Child.*, **52**, 525.

EVANS, A.E., GILBERT, E.S. & ZANDSTRA, R. (1970). Increasing incidence of central nervous system leukaemia in children. *Cancer*, **26**, 404.

GEORGE, S.L., OCHS, J.L., MAUER, A.M. *et al.* (1985). The importance of an isolated CNS relapse in children with acute lymphoblastic leukaemia. *J. Clin. Oncol.*, **3**, 776.

GREEN, D.M., FREEMAN, A.L., SATHER, H.N. *et al.* (1980). Comparison of three methods of central nervous system prophylaxis in childhood acute lymphoblastic leukaemia. *Lancet*, **i**, 1398.

HARDISTY, R.M. & NORMAN, P.M. (1967). Meningeal leukaemia. *Arch. Dis. Child.*, **42**, 441.

HOLLAND, J.F. & GLIDEWELL, O. (1972). Chemotherapy of acute lymphocytic leukaemia of childhood. *Cancer*, **30**, 1480.

HUSTU, H.O., AUR, R.J.A., VERZOSA, M.S. *et al.* (1973). Prevention of central nervous system leukaemia by irradiation. *Cancer*, **32**, 585.

JANNOUN, L. (1983). Are cognitive and education development affected by age at which prophylactic therapy is given in acute lymphoblastic leukaemia? *Arch. Dis. Child.*, **58**, 953.

LAND, B.J., THOMAS, P.R.M., BOYETT, J.M. *et al.* (1985). Comparison of maintenance treatment regimes for first CNS relapse in children with acute lymphoblastic leukaemia: a paediatric oncology group study. *Cancer*, **56**, 81.

NESBIT, M.E., SATHER, H.N., ROBISON, L.L. *et al.* (1981). Presymptomatic CNS therapy in previously untreated childhood acute lymphoblastic leukaemia comparison of 1800 rads and 2400 rads. *Lancet*, **i**, 461.

ORTEGA, J.A., NESBIT, M.E., SATHER, H.N. *et al.* (1987). Long term evaluation of a CNS prophylaxis trial – treatment comparison and outcome after CNS relapse in childhood ALL. A report from the Children's Cancer Study Group. *J. Clin. Oncol.*, **5**, 1646.

POPLACK, D., REAMAN, G., BLEYER, A. *et al.* (1984). Central nervous system preventive therapy with high dose methotrexate in acute lymphoblastic leukaemia. *Proc. Am. Soc. Clin. Oncol.*, **3**, 204.


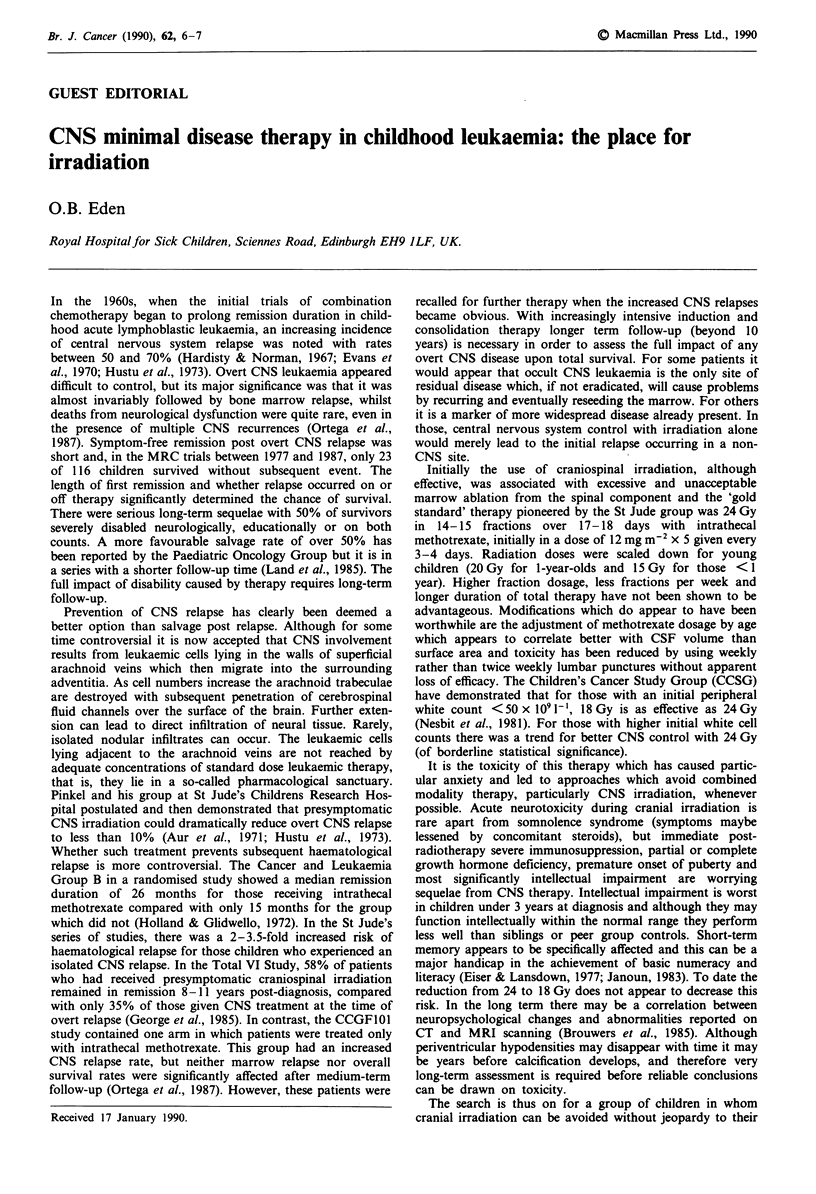

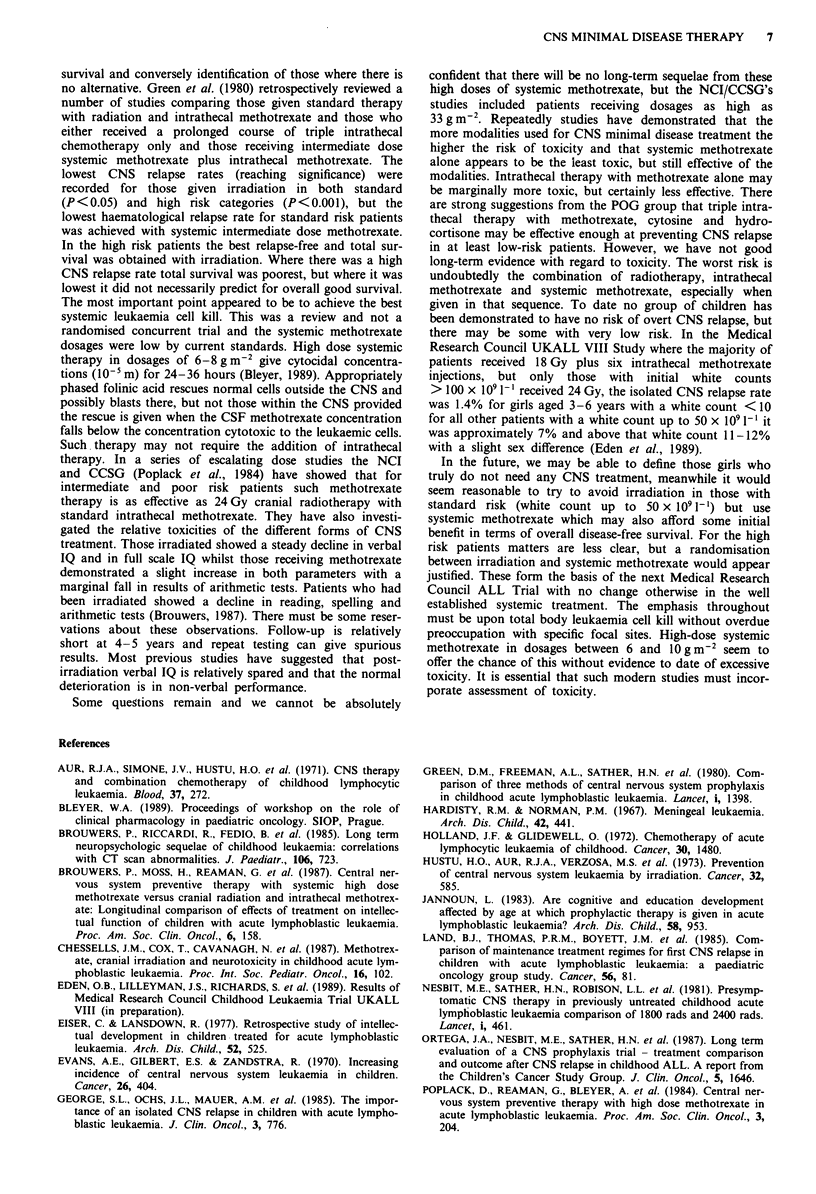


## References

[OCR_00237] Aur R. J., Simone J., Hustu H. O., Walters T., Borella L., Pratt C., Pinkel D. (1971). Central nervous system therapy and combination chemotherapy of childhood lymphocytic leukemia.. Blood.

[OCR_00246] Brouwers P., Riccardi R., Fedio P., Poplack D. G. (1985). Long-term neuropsychologic sequelae of childhood leukemia: correlation with CT brain scan abnormalities.. J Pediatr.

[OCR_00269] Eiser C., Lansdown R. (1977). Retrospective study of intellectual development in children treated for acute lymphoblastic leukaemia.. Arch Dis Child.

[OCR_00274] Evans A. E., Gilbert E. S., Zandstra R. (1970). The increasing incidence of central nervous system leukemia in children. (Children's Cancer Study Group A).. Cancer.

[OCR_00279] George S. L., Ochs J. J., Mauer A. M., Simone J. V. (1985). The importance of an isolated central nervous system relapse in children with acute lymphoblastic leukemia.. J Clin Oncol.

[OCR_00284] Green D. M., Freeman A. I., Sather H. N., Sallan S. E., Nesbit M. E., Cassady J. R., Sinks L. F., Hammond D., Frei E. (1980). Comparison of three methods of central-nervous-system prophylaxis in childhood acute lymphoblastic leukaemia.. Lancet.

[OCR_00289] Hardisty R. M., Norman P. M. (1967). Meningeal leukaemia.. Arch Dis Child.

[OCR_00293] Holland J. F., Glidewell O. (1972). Chemotherapy of acute lymphocytic leukemia of childhood.. Cancer.

[OCR_00297] Hustu H. O., Aur R. J., Verzosa M. S., Simone J. V., Pinkel D. (1973). Prevention of central nervous system leukemia by irradiation.. Cancer.

[OCR_00302] Jannoun L. (1983). Are cognitive and educational development affected by age at which prophylactic therapy is given in acute lymphoblastic leukaemia?. Arch Dis Child.

[OCR_00307] Land V. J., Thomas P. R., Boyett J. M., Glicksman A. S., Culbert S., Castleberry R. P., Berry D. H., Vats T., Humphrey G. B. (1985). Comparison of maintenance treatment regimens for first central nervous system relapse in children with acute lymphocytic leukemia. A Pediatric Oncology Group study.. Cancer.

[OCR_00313] Nesbit M. E., Sather H. N., Robison L. L., Ortega J., Littman P. S., D'Angio G. J., Hammond G. D. (1981). Presymptomatic central nervous system therapy in previously untreated childhood acute lymphoblastic leukaemia: comparison of 1800 rad and 2400 rad. A report for Children's Cancer Study Group.. Lancet.

[OCR_00319] Ortega J. A., Nesbit M. E., Sather H. N., Robison L. L., D'Angio G. J., Hammond G. D. (1987). Long-term evaluation of a CNS prophylaxis trial--treatment comparisons and outcome after CNS relapse in childhood ALL: a report from the Childrens Cancer Study Group.. J Clin Oncol.

